# Supplementary material for: Inflammatory response in hematopoietic stem and progenitor cells triggered by activating SHP2 mutations evokes blood defects
Source: eLife. 2022 May 10;11:e73040. doi: 10.7554/eLife.73040 (PMC9119675; doi:10.7554/eLife.73040)

Figure 1-source data 1
Uncropped and unedited blots for the associated panel B in Figure 1.The red boxed indicate the cropped regions.

Total Shp2

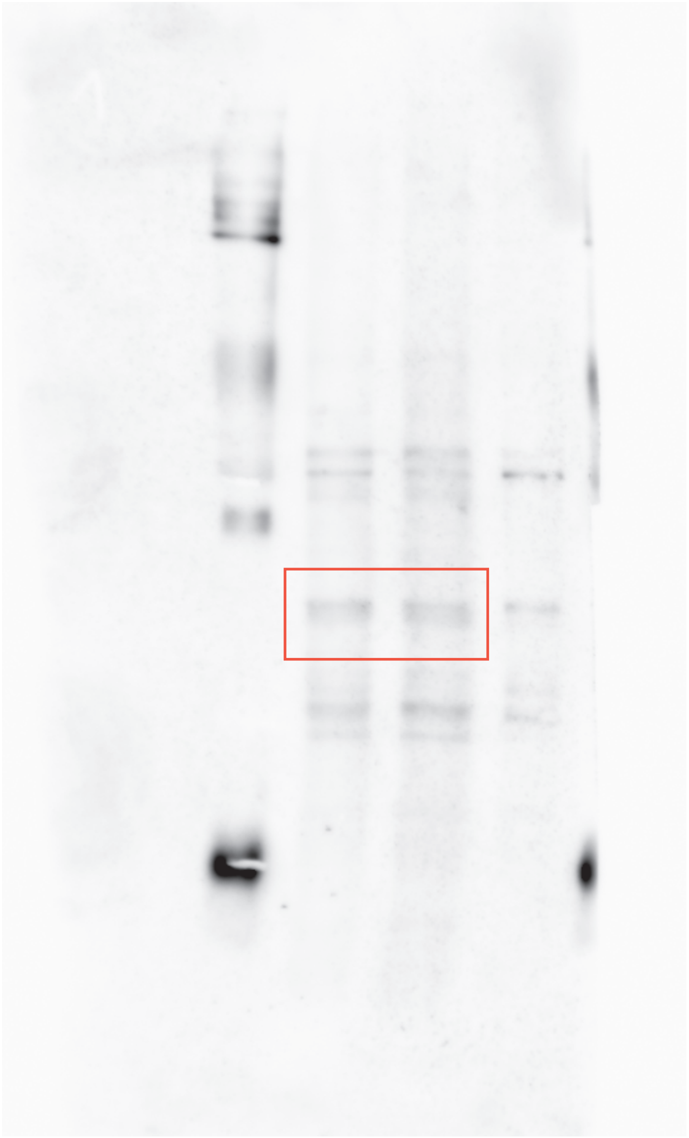

tubulin

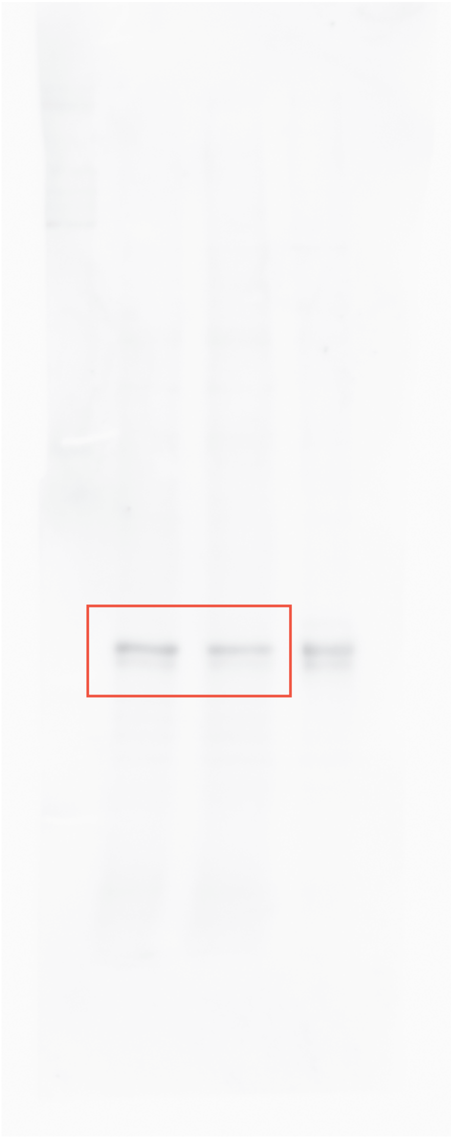

Supplement: Figure 1—source data 1. [file elife-73040-fig1-data1.pdf]
